# Pharmacokinetics and pharmacodynamics of cannabigerol (CBG) in the C57BL/6Crl mouse

**DOI:** 10.3389/fphar.2025.1672098

**Published:** 2025-12-09

**Authors:** Ayat Zagzoog, Kenzie Halter, Nini Ha, Alayna M. Jones, Rachel Andres, Andy Kim, Deborah Michel, Jane Alcorn, Robert B. Laprairie

**Affiliations:** 1 College of Pharmacy and Nutrition, University of Saskatchewan, Saskatoon, SK, Canada; 2 College of Medicine, University of Saskatchewan, Saskatoon, SK, Canada

**Keywords:** cannabigerol, intoxication, pharmacokinetics, pharmacodynamics, cannabinoids

## Abstract

**Introduction:**

Cannabis holds therapeutic potential; however, activation of the type 1 cannabinoid receptor (CB1R) via Δ^9-^tetrahydrocannabinol (THC) is also responsible for the characteristic “high” induced by cannabis. The pharmacology of the less abundant phytocannabinoid, cannabigerol (CBG), is poorly established, though it has been shown to exhibit promising therapeutic properties such as potential anxiolytic effects.

**Methods:**

We assessed the pharmacokinetics (PK) and pharmacodynamics (PD) of CBG in C57BL/6Crl mice, hypothesizing that CBG would produce fewer PD effects than we had previously observed with THC, even when accounting for PK differences. Following oral (*p.o*.), intraperitoneal (*i.p*.), and intravenous (*i.v.*) administration, the PK profile of CBG was assessed via blood sampling at specified time points (10 min, 30 min, 1 h, 3 h, 6 h, 12 h, 18 h, and 24 h). The blood concentrations of CBG were quantified by High-Performance Liquid Chromatography-Tandem Mass Spectrometry (HPLC-MS/MS). A separate cohort of mice was treated with CBG and tested for cataleptic, hypothermic, anti-nociceptive, and locomotor effects to correlate the PK profile of CBG with CBG’s observed PD effects.

**Results and Discussion:**

Our data reveal that CBG was not intoxicating, even when accounting for the route of administration and blood concentration. Our findings support previous reports that CBG is not intoxicating and reveal that even if CBG were present at sufficiently high concentrations in cannabis products, it would not produce intoxicating effects like those of THC.

## 1 Introduction

Δ^9^-Tetrahydrocannabinol (THC) is known to be a CB1R partial agonist that produces the characteristic “high” associated with cannabis, as well as therapeutic pain- and anxiety-modulating effects (Maccarrone et al., 2023). The pharmacology of the less-abundant phytocannabinoid, cannabigerol (CBG), is not as established as THC, but CBG has been described as a CB1R partial agonist with an affinity of approximately one-twentieth that of THC (El-Alfy et al., 2010; Navarro et al., 2018; Zagzoog et al., 2020). CBG is also a CB2R agonist, which may explain CBG’s potential anti-inflammatory and immunomodulatory effects (Anil et al., 2022; Lowin et al., 2023). CBG may be responsible for some of the anti-anxiety effects of cannabis (reviewed in Calapai et al., 2022) and reduces the severity of Huntington’s disease and Parkinson’s disease in animal models (Nachnani et al., 2021; Calapai et al., 2022). In our own work, we observed that *i.p.*-administered CBG reduced nociception and anxiety in male mice, but we did not explicitly determine the receptor mechanism (Zagzoog et al., 2020). Due to its low *in vitro* affinity for CB1R, CBG is often considered a non-psychoactive phytocannabinoid; however, *in vivo* studies supporting this contention are lacking (reviewed in Jastrząb et al., 2022).

CBG interacts with a variety of receptors beyond the cannabinoid receptors. CBG is an agonist of α_2_-adrenoceptor and the serotonin 1A receptors (5-HT1A), which are involved in regulating mood, anxiety, and depression (Cascio et al., 2010; Mendiguren et al., 2023; Rock et al., 2011). CBG is a transient receptor potential vanilloid 1 (TRPV1) receptor agonist and transient receptor potential ankyrin 1 (TRPA1) receptor antagonist, both of which play roles in pain perception and inflammation (De Petrocellis et al., 2008; De Petrocellis et al., 2011). CBG also acts as an antagonist at the GPR55 receptor, which is implicated in pain, inflammation, and cancer (Navarro et al., 2018). Furthermore, CBG exhibits agonist activity at peroxisome proliferator-activated receptors (PPARs), which are involved in the regulation of metabolism, inflammation, and cellular differentiation (Granja et al., 2012). These diverse interactions underscore CBG’s complex pharmacology.

The physicochemical properties of CBG are like other cannabinoids, indicating that CBG is likely to share similar PK properties with THC (reviewed by Millar et al., 2018). A study in male Swiss mice identified that CBG’s limited oral bioavailability is due to extensive first-pass metabolism and poor water solubility. This was further confirmed with higher brain concentrations of CBG following *i.p.* administration relative to *p.o.* administration (Deiana et al., 2012). The rate of absorption was rapid, with time to peak plasma concentration (T_max_) at 30 min for *p.o.* administration and 2 h for *i.p.* administration (Deiana et al., 2012). In frequent cannabis smokers, CBG was detected up to 17 min after smoking and up to 13 min following vaporization (Newmeyer et al., 2016). When cannabis is administered orally, CBG was not detected in the blood of subjects at the dose given (Newmeyer et al., 2016). Whereas THC and cannabidiol (CBD) are primarily metabolized in the liver by cytochrome P450 enzymes (Jiang et al., 2013), the specific drug metabolism pathways and metabolites of CBG require further investigation. Although the half-life of CBG in humans has not been extensively studied, it is anticipated to have a long half-life owing to its lipophilic properties and propensity for tissue accumulation. CBG is expected to be eliminated primarily by hepatic metabolism, and its metabolites are excreted primarily through feces and, to a lesser extent, through the urine, similar to THC (reviewed in Jastrząb et al., 2022).

**FIGURE 1:**
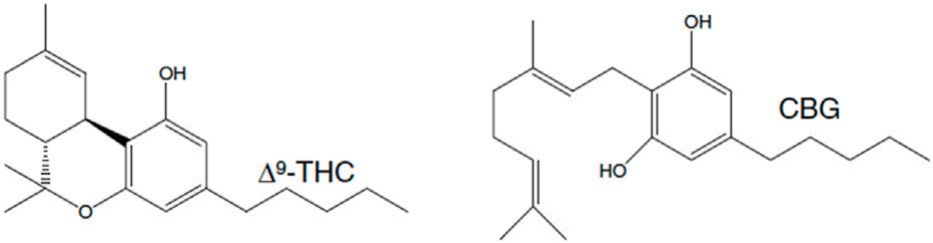
Molecular structures of phytocannabinoids used in this study. Structures were drawn by ChemSketch.

In this study, the pharmacokinetics (PK) and pharmacodynamics (PD) of CBG were evaluated following a single intravenous (*i.v.*), intraperitoneal (*i.p.*), or oral (*p.o.*) administration to determine whether CBG has any intoxicating properties when accounting for route of administration, dose, sex, or blood concentration. We hypothesized that CBG would produce an intoxicating effect in C57BL/6Crl mice that was lower in magnitude than that of our previous observations with THC (Figure 1) (Zagzoog et al., 2024). To test this hypothesis, blood concentration *versus* time profiles were generated for CBG following *p.o., i.p.,* and *i.v.* administration in male mice. These data were used to estimate PK parameters (C_max_, t_1/2,_ AUC, V_d_, Cl_S_, k, and F). Assessments of physiological responses to CBG were made at the time corresponding to the highest measured blood concentration of CBG via *i.v., i.p.,* or *p.o.* routes. The described research provides data on how CBG’s pharmacology differs from THC and whether this difference is of medicinal importance or significant in the context of safe use.

## 2 Materials and methods

### 2.1 Materials

CBG (Cat. No. 15293) and THC (Cat. No. 12068) were purchased from Cayman Chemical (Ann Arbor, Michigan 48108 United States) and stored at −20 °C. Analytical standards CBG (Cat. No. C-141) and structure analogue deuterated internal standards CBD-d3 (Cat. No. C-084) were purchased from Cerilliant (Round Rock, TX, United States). Liquid chromatography-mass spectrometry (LC-MS)–grade methanol, water, acetonitrile, formic acid, and ammonium formate were purchased from Thermo Scientific (Waltham, MA, United States). All other materials, such as ethanol, Kolliphor, phosphate-buffered saline, DMSO, and HybridSPE®-Phospholipid 96-well plates, were from Sigma-Aldrich (Mississauga, ON).

### 2.2 Animals

Adult male and female C57BL/6Crl mice aged 8–12 weeks were purchased from Charles River Labs (Senneville, QC). Animals were group housed (5 females per cage and 3 males per cage), with *ad libitum* access to food, water, and environmental enrichment, and were maintained on a 12 h light/dark cycle at the Laboratory Animal Services Unit (LASU) at the University of Saskatchewan. All protocols were in accordance with the guidelines detailed by the Canadian Council on Animal Care and approved by the Animal Research Ethics Board and the Scientific Merit Review Committee for Animal Behaviour at the University of Saskatchewan (AUP #20200043) (Percie du Sert et al., 2020; Zagzoog et al., 2024). The animals had a period of acclimatization without any handling for 7 days, followed by an additional 7–10 days with handling before the start of the experiment (CBG, n = 136 total mice; THC, n = 48 mice). All samples collected from animals were stored in protein LoBind® Eppendorf tubes (Fisher, Ottawa, ON) to limit analyte loss from plastic binding. Intravenous (*i.v.*) treatment was performed by tail vein injection; *i.p.* injection was performed in the lower right or left quadrant of the mouse’s abdomen; and oral gavage (*i.e., p.o.*) was performed with a 20G, curved, mouse feeding needle.

### 2.3 Study phase I

**FIGURE 2:**
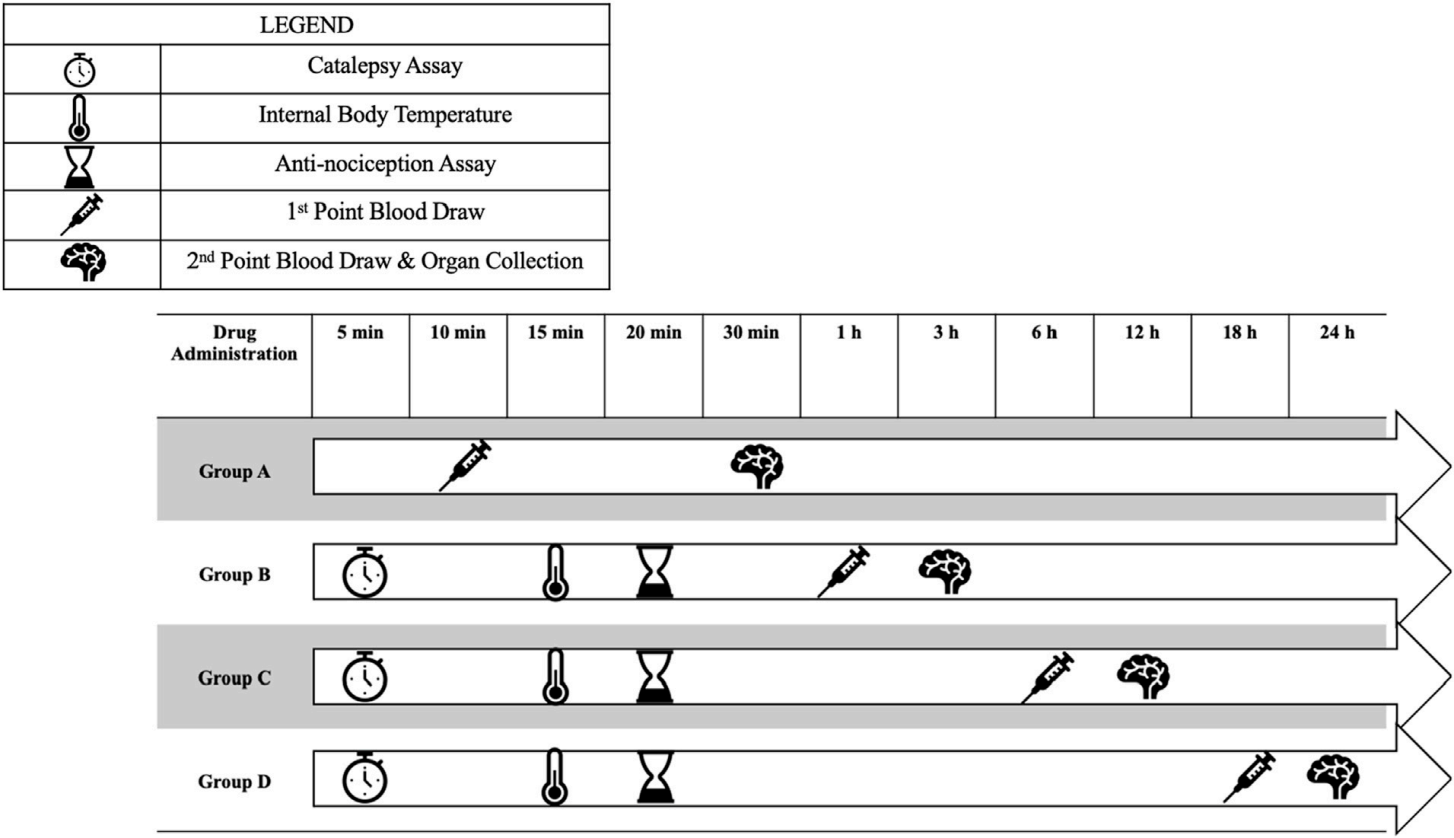
Timeline representation for pharmacokinetic and pharmacodynamic evaluations. Mice proceeded through the experiment for all groups except A, followed by blood collection via the saphenous vein and cardiac puncture, respectively. Figure created by the author in PowerPoint.

In phase I, male mice were randomly assigned to Groups A, B, C, or D. Interventions per group are specified in Figure 2. Each group was divided into 3 cohorts and randomly assigned to receive *p.o., i.p.,* or *i.v.* administration. Oral (*p.o*.) administration cohorts received 10 mg/kg CBG in olive oil with 10% DMSO, and *i.p.* and *i.v.* cohorts received 10 mg/kg CBG with 10% DMSO and vehicle (1:1:18 ethanol: kolliphor: saline). PK profiles were developed for blood collection at 10 min, 30 min, 1 h, 3 h, 6 h, 12 h, 18 h, and 24 h. Blood collection was performed using a destructive sampling method, with each mouse providing blood at two time point collections. The initial blood draw aimed to obtain a 100 µL blood sample from the left leg lateral saphenous vein of the mouse, and the second blood draw from the same mouse involved the administration of isoflurane (3%–5%) followed by exsanguination via cardiac puncture. Group A was subjected to two time point blood draws, at 10 min and at 30 min. Groups B-D were first subjected to the cannabinoid-induced tetrad assay, then two time point blood draws. Due to time constraints, the open field test (OFT) was not performed in Phase I.

Blood collection from the saphenous leg vein was drawn into a RAM Scientific Safe-T-Fill™ Capillary blood collection system containing heparin and lithium (Waltham, Massachusetts, U.S). Blood collection from the cardiac puncture was drawn into a syringe and transferred to a 2 mL blood collection tube containing heparin and lithium. Samples were immediately placed on ice, transferred to LoBind® tubes as quickly as possible, and then stored at −80 °C. The blood concentrations of CBG were determined using High-Performance Liquid Chromatography-Tandem Mass Spectrometry (HPLC-MS/MS).

The PK parameters for CBG were determined by GraphPad Prism (v. 10.2.3). Linear regression analysis natural log of the concentration *versus* time curve was utilized to derive the terminal phase rate constant (*k*), from which the terminal phase half-life (t_1/2_) was calculated using the ratio (0.693/*k*). For both *p.o.* and *i.p.* administration, the C_max_ was obtained directly from visual inspection of the blood concentration *versus* time profiles. For the purposes of this study, T_max_ refers to the time at which C_max_ was reported. The log-linear trapezoidal method was used to calculate the total area under the curve (AUC) of the average blood concentrations *versus* time profile with extrapolation to infinity, where the tail of the AUC (i.e., last concentration extrapolated to infinity) was calculated as the ratio C_p,last_/k. Bioavailability was estimated by dividing the mean total AUC_∞_ for each route of administration by the total AUC observed for each compound following *i.v.* administration (*where* F = 100%, by definition). Systemic clearance (Cl_S_) was calculated as the ratio Dose_*i.v.*_/AUC  ∞ . The apparent volume of distribution (V_d_) was calculated as Dose_*i.v.*_/(k × AUC  ∞ ).

#### 2.3.1 Cannabinoid-induced tetrad

The cannabinoid-induced tetrad includes four phenotypic effects of CB1R agonists: catalepsy (an impaired capacity to initiate movement); hypothermia; anti-nociception (a decrease of pain sensitivity); and hypo-locomotion (a decrease of spontaneous horizontal activity) (Grim et al., 2017; Metna‐Laurent et al., 2017). Assessments were conducted in Phase I (Parts A and B) and Phase II following drug administration at the times indicated in the figure legends.

Catalepsy was assessed in the ring holding assay; mice were positioned with their forepaws grasping onto a 0.5 cm thick ring clamp elevated 5 cm above the testing surface. The duration of time while the ring was held was recorded in seconds, with each trial terminated if the mouse’s forepaw released from the bar, upon turning of the head or body, or after 60 s of immobility. Catalepsy was evaluated three times consecutively, and the average time spent holding the bar was reported as a percent of the maximum possible effect (% MPE, up to 60 s). Internal body temperature was measured via a rectal thermometer. Nociception was determined by assessing tail-flick latency, where mice were restrained with their tails placed ∼1 cm into 52 °C ± 2 °C water. The time (sec) until the tail was removed was measured, with observations ending at 20 s and represented as a % MPE. Locomotion and anxiety-like behaviour were assessed in the OFT. Mice were placed in an open space (90 cm × 90 cm) and recorded for 5 min. Total distance travelled (cm), velocity (cm/sec), time in the centre quadrant (sec), and central quadrant entries were measured with EthoVision XT (Noldus Information Technology Inc., Leesburg, VA).

In Phase I (Part B), after determining the timepoint at which blood CBG concentrations were highest for each respective route of administration, the timing for each measure in the tetrad was adjusted. An assessment was conducted in a new cohort consisting of both male and female mice (n = 4 per sex per route) receiving a dose of 10 mg/kg of CBG via *i.v.*, *i.p.*, or *p.o.* routes. For those administered with *i.v.* CBG, the timing was: catalepsy at 5 min, body temperature at 15 min, tail-flick latency at 20 min, and OFT at 25 min post-injection. For *i.p.* CBG, measurements were taken at 20 min for catalepsy, 25 min for body temperature, 30 min for tail-flick latency, and 35 min for OFT post-injection. For *p.o.* CBG, data collection was scheduled as follows: catalepsy at 170 min, body temperature at 175 min, tail-flick latency at 180 min, and OFT at 185 min post-injection.

### 2.4 Study phase II

In phase II, male and female mice were randomly assigned to different treatment cohorts. Four different dose cohorts per sex were each randomly assigned to receive 1, 3, 10, or 30 mg/kg *i.v.* CBG. Four animals from each cohort were randomly assigned to undergo cannabinoid tetrad assessment, while the remaining 4 in each cohort underwent cardiac puncture for blood collection at the anticipated highest blood compound concentration. Blood collected from the cardiac puncture was drawn into a syringe and then transferred to a 2 mL blood collection tube containing heparin and lithium, immediately placed on ice, then transferred to LoBind® tubes as quickly as possible, and stored at −80 °C. Blood concentrations were determined as described below using HPLC-MS/MS. To assess CBG activity relative to THC, additional calculations of THC intoxication equivalency were determined for potency for each measure of the tetrad (catalepsy, hypothermia, and anti-nociception).

### 2.5 Quantitative analysis using HPLC-MS/MS

HPLC-MS/MS was used to quantify CBG in the blood using a 1,260 Agilent (Agilent Technologies Canada, Mississauga, ON) High Performance Liquid Chromatography (HPLC) system connected to an AB Sciex 4,000 Hybrid Triple Quadruple Linear Ion Trap Mass Spectrometer (QTRAP) equipped with a Turboionspray™ interface (AB Sciex, Concord, ON). The analytical method was developed and validated for mouse blood. An Infinity lab Phenyl Hexyl column (4.6 × 12.5 mm, 5 μm) and guard column (4.6 × 12.5 mm, 5 μm) were used to separate compounds, and the temperature was set to 50 °C.

A volume of 5 μL was injected onto the column using a 1,260 Agilent autoinjector set to 4 °C. Mobile phases A and B consisted of water and methanol, spiked with 1 mM of ammonium formate and 0.1% formic acid, respectively. The mobile phase flow rate was 500 μL/min, starting with an isocratic hold for 0.1 min with 85% B, then switched to 95% B in a gradient over 5 min, held isocratic at 95% B to 7 min. The gradient returned to a 15:85 ratio for a total running time of 7.1 min–10 min.

Multiple reaction monitoring (MRM) was achieved by using electrospray ionization (ESI) in positive ion mode. The monitored precursor ion and product ion transitions for CBG were 317.18 → *m/z* 193.1 and 317.18 → *m/z* 123.1, and for structural analog internal standard CBD-d3 318.25 → *m/z* 196.1. The source temperature was set to 700 °C, ion spray voltage (ISV) 3500V, curtain gas (CUR) 40, nebulizer gas (GS1) 40, heater gas (GS2) 60, collision gas (CAD) medium, and using an exit potential of 10 for all transitions. The Dwell time for all transitions was 50 msec at unit resolution. Nitrogen was used as the gas, and the interface heater was on for all cases. ABSciex Analyst 1.7 was used for data acquisition and analysis.

The Food and Drug Administration (FDA) and European Medicines Agency (EMA) Guidance for Bioanalytical Method Validation (2018) (US Department of Health and Human Services Food and Drug Administration Center for Drug Evaluation and Research and Center for Veterinary Medicine, 2018), including matrix effects, selectivity, carry-over, linearity, precision, accuracy, recovery, reproducibility, and stability were followed for method validation; details are found within the Supplementary Data for this manuscript (Supplementary Figures S1 and S2; Supplementary Tables S1–S3).

### 2.6 Statistical analysis

Collected blood was subject to HPLC-MS/MS analysis to quantify cannabinoid levels, and any data points that fell below the lowest limit of quantification (LLOQ, 8 ng/mL) were excluded. Data were expressed as the mean ± standard error of the mean (SEM) or 95% confidence interval (CI), as indicated in the Table and Figure legends. Variation within each dataset is presented by plotting data points representing individual samples. Physiological data are presented as % of maximum possible effect (MPE) for catalepsy (MPE = 60 s) and anti-nociception (MPE = 20 s), °C for body temperature, cm for distance travelled, cm/sec for velocity, and seconds for time in the centre quadrant. Statistical analyses were conducted using one-sample *t*-tests or one- or two-way analysis of variance (ANOVA) with GraphPad Prism (v. 10.2.3), as indicated in figure legends. To reduce animal use, for one sample *t*-tests of data presented in Figures 4, 5, data were compared to 1% (catalepsy), 37.9 °C (body temperature), and 6.5% (tail flick latency) based on mean values previously observed for vehicle treatment (Zagzoog et al., 2024). Post-hoc analyses were performed using Tukey’s test. Post-hoc analyses of one-way ANOVAs included multiple comparisons between all groups; and *post hoc* analyses of two-way ANOVAs included multiple comparisons between sexes within treatment, between sexes within dose, between routes within treatment, or between treatments within route, with groups defined in each figure legend. All dose-response data were fitted to a three-parameter non-linear regression analysis to estimate potency (ED_50_). To estimate drug potency related to THC while considering blood drug concentration at the highest cannabinoid blood levels, drug equivalency ratios were calculated potency for each measure of the tetrad (Equations 1, 2). THC potency and blood concentrations, and vehicle treatment data in Figures 4, 5, are from Zagzoog et al. (2024), as experiments in these studies were conducted simultaneously. Estimates from Equations 1, 2 were used to produce an equivalency ratio compared to THC (Equation 3).

$$THC\;potency:\frac{{ED}_{50}\;\left(\frac{mg}{mL}\right)}{\left[THC\right]\left(\frac{ng}{mL}\right)}$$ (1)

$$CBG\;potency:\frac{{ED}_{50}\;\left(\frac{mg}{mL}\right)}{\left[CBG\right]\left(\frac{ng}{mL}\right)}$$ (2)

$$Potency\;ratio:\frac{THC\;potency}{CBG\;potency}$$ (3)

**FIGURE 3:**
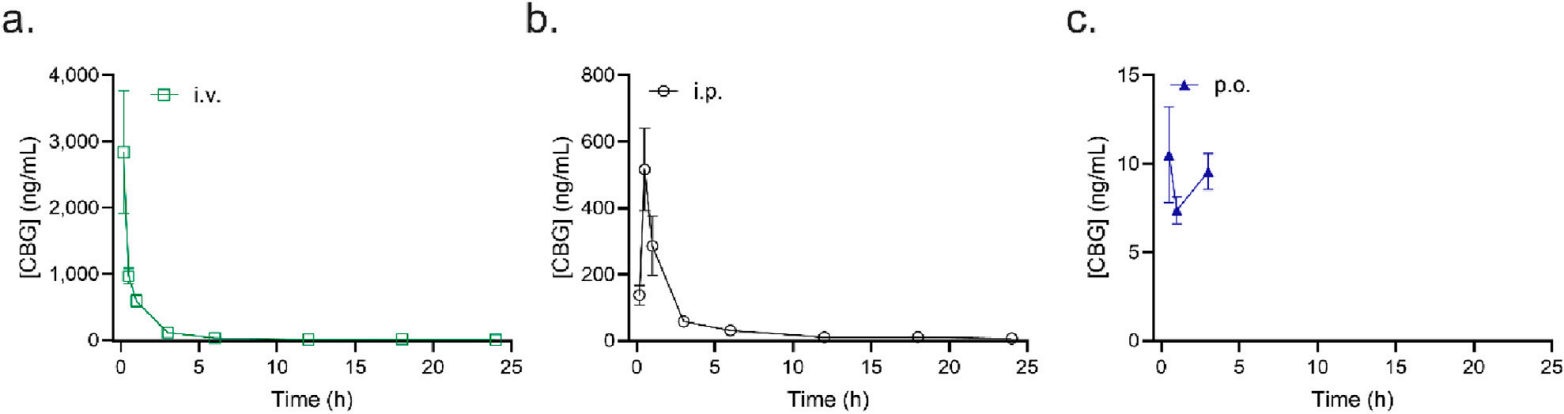
Mean ± SEM blood CBG concentration vs. time curves following a single bolus dose of 10 mg/kg of CBG given by intravenous (*i.v.)* **(a)** *,* intraperitoneal *(i.p.)* **(b)**, or oral (*p.o*.) **(c)** route of administration. Male mice aged 8–12 weeks were administered CBG, and blood was collected using a destructive sampling approach where each mouse provided blood at two time points, with n = 4 mice/time point according to the *Phase I Pharmacokinetic Study Intervention Timeline* (Figure 2). Each group was divided into 3 cohorts, where each mouse was randomly assigned either *i.v., i.p., or p.o*. drug administration. *Note,* y-axes vary between panels to better display the data.

**FIGURE 4:**
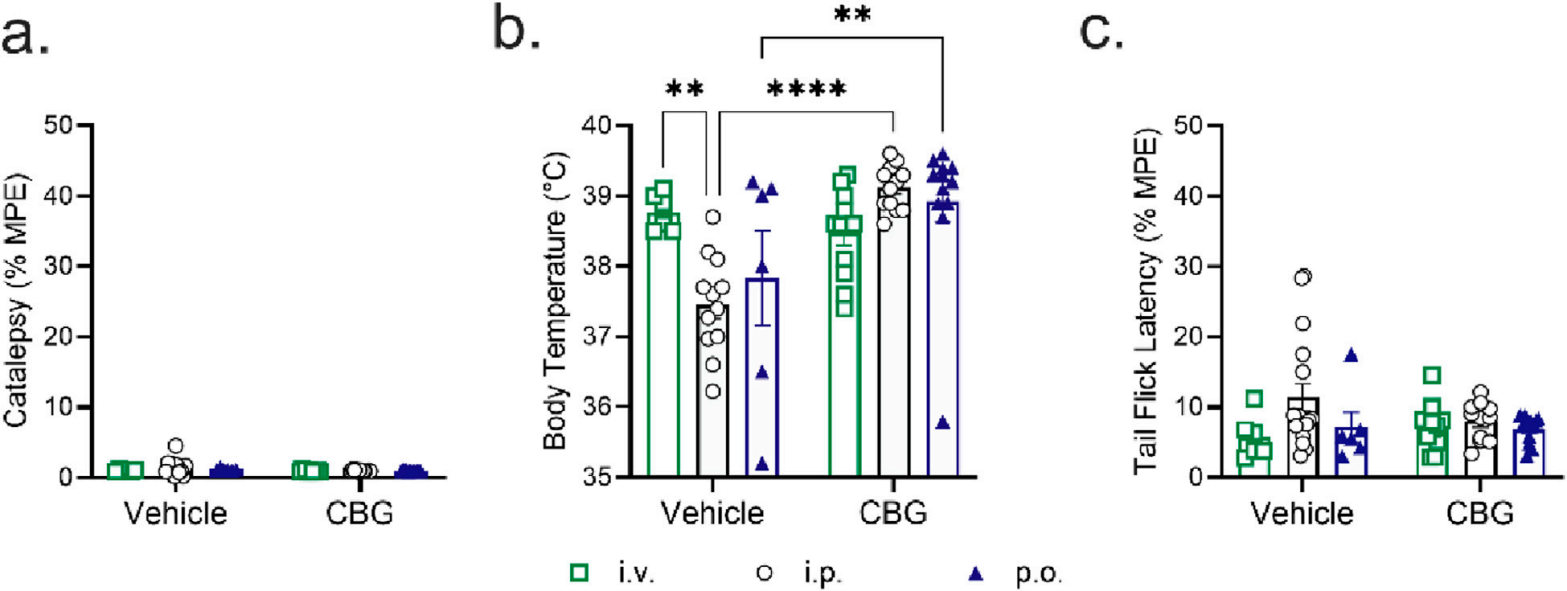
Acute tetrad effects in male C57BL/6Crl mice following a single bolus dose of 10 mg/kg of CBG given by intravenous (*i.v.),* intraperitoneal *(i .p.)*, or oral (*p.o*.) route of administration (n = 6–17 mice/route of administration; *note* *i.p.* for tail flick latency one datapoint was excluded due to a recording instrument failure). Male mice aged 8–12 weeks were administered CBG and assessed for **(a)** catalepsy 5 min post-injection; **(b)** body temperature 15 min post-injection and **(c)** anti-nociception in the tail-flick assay 20 min post-injection. Catalepsy data are expressed as the % maximum possible effect (MPE = 60 s). Body temperature data are expressed as the measured body temperature via rectal thermometer (°C). Tail-flick latency data are expressed as the % maximum possible effect (MPE = 20 s). Data are expressed as mean ± SEM. ****p < 0.0001, **p < 0.01 as determined by two-way ANOVA (route *x* treatment) followed by Tukey’s *post hoc* test. *Note*, vehicle data were previously included in Zagzoog et al. (2024); and the scale of y-axes varies between panels.

**FIGURE 5:**
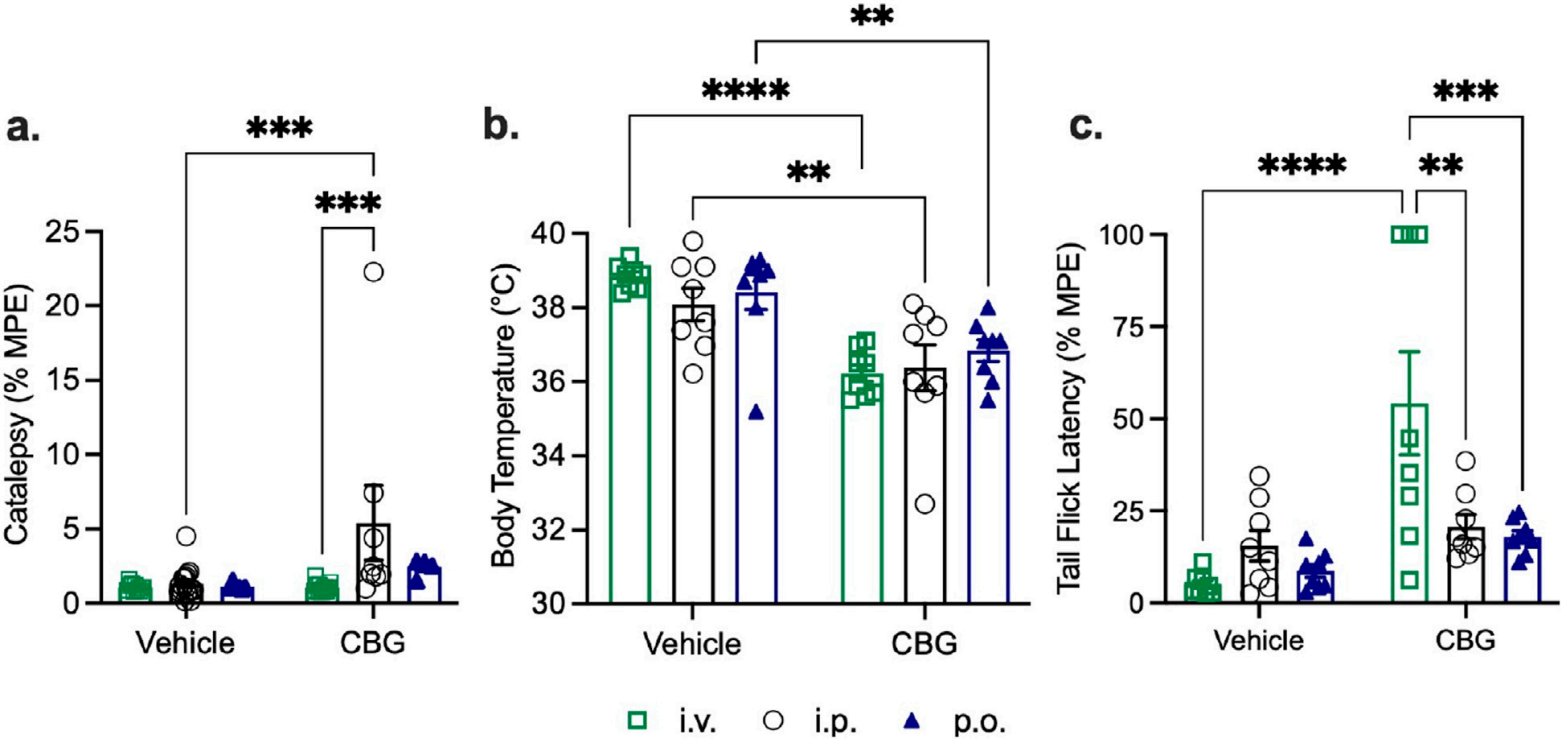
Physiological effects of 10 mg/kg of CBG in male and female C57BL/6Crl mice. Data are presented as combined sex for each route of administration, intravenous (*i.v.),* intraperitoneal *(i.p.)*, or oral (*p.o*.) (n = 8–16 per group). **(a)** Catalepsy time was 5 min post-injection for *i.v*., 20 min post-injection for *i.p*., and 170 min post-injection for *p.o*. **(b)** Body temperature was measured at 15 min post-administration for *i.v.*, 25 min post-administration for *i.p*., and 175 min post-administration for *p.o.*. **(c)** Nociception in the tail-flick latency test was 20 min post-administration for *i.v.,* 30 min post-administration for *i.p.*, and 180 min post-administration for *p.o.*. Data for catalepsy are represented as % maximum possible effect (MPE) during a maximum of 60 s. Catalepsy data are expressed as the % maximum possible effect (MPE = 60 s). Body temperature data are expressed as the measured body temperature via rectal thermometer (°C). Tail-flick latency data are expressed as the % maximum possible effect (MPE, = 20 s). Data are expressed as mean ± SEM. ****p < 0.0001, ***p < 0.001, and **p < 0.01 as determined by two-way ANOVA (route *x* treatment) followed by Tukey’s *post hoc* test. *Note*, vehicle data were previously included in Zagzoog et al. (2024); and the scale of y-axes varies between panels.

## 3 Results

### 3.1 Study phase I

#### 3.1.1 Pharmacokinetic profiles

Male mice received 10 mg/kg of CBG by *i.v., i.p.,* or *p.o*. administration, and blood samples were collected at 10 min, 30 min, 1 h, 3 h, 6 h, 12 h, 18 h, and 24 h post-administration. The blood concentrations of CBG *versus* time profiles suggested CBG exhibits multi-compartmental kinetics (Figure 3). Intravenous and extravascular PK parameters calculated using noncompartmental methods are reported in Table 1. Intraperitoneal administration of 10 mg/kg CBG gave a C_max_ of 516 ng/mL at 30 min, whereas we had previously observed that *i.p.* dosing with 10 mg/kg THC reached a C_max_ of 377 ng/mL at 10 min (Zagzoog et al., 2024). In comparison, *p.o.* CBG resulted in a C_max_ (10.5 ng/mL) that was 2% of *i.p.* CBG and did not occur until 180 min; by comparison, we previously found the THC *p.o.* C_max_ (149.5 ng/mL) was 40% that of *i.p.* THC and was observed 60 min after administration (Zagzoog et al., 2024). The terminal rate constants (*k*) of *i.v.* and *i.p.* CBG were similar (Table 1). The terminal rate constant (*k*), t_1/2_, and bioavailability (F) for *p.o.* administration of CBG could not be estimated from the current dataset due to insufficient data (at many time points, CBG blood concentrations were below the LLOQ). Following *i.p.* administration, the absolute bioavailability of CBG was 0.4 (40%), which was lower than the 60% we previously reported for THC (Zagzoog et al., 2024).

**TABLE 1:** Pharmacokinetic parameter estimates following a single bolus dose administration of 10 mg/kg CBG given by intravenous (*i.v.*), intraperitoneal (*i.p*.), or oral (*p.o*.) routes of administration in male C57BL/6Crl mice aged 8–12 weeks. Data presented in Figure 3 **.**

Pharmacokinetic parameters	CBG (10 mg/kg)		
	*i.v.*	*i.p.*	*p.o.*
*k* (h^-1^)	0.0698	0.069	c.n.d.
*α* (h^-1^)	1.016	–	–
t_1/2_ (h)	9.9	10.0	c.n.d.
*C*_max_ (ng/mL)	–	516	10.5
*T*_max_ (min)	–	30	180
AUC_0-_ ∞	2,804	1,169	c.n.d
F	–	0.4	c.n.d.
Cl_S_ (mL/min/kg)	59.43	–	c.n.d.
V_d_ (mL/kg)	51,100	–	c.n.d.

Data are expressed as a pooled estimate of male mice blood levels, where each mouse provided blood for two time points, with 4 mice/time point. PK, parameters were estimated using noncompartmental analysis. c. n.d, could not be determined; *i.v*., intravenous; *i.p*., intraperitoneal; *p.o*., per os or oral.

#### 3.1.2 Initial physiological analyses not accounting for the expected T_max_

Male C57BL/6Crl mice aged 8–12 weeks were treated with 10 mg/kg of CBG, followed by an assessment for catalepsy, hypothermia, and anti-nociception (Figure 4). The *i.v., i.p.*, and *p.o*. routes of CBG administration did not produce a cataleptic response (Figure 4a). A significant increase in temperature was noted in CBG-treated mice via *i.p.* or *p.o.* routes compared to vehicle (Figure 4b), which is the opposite of the expected hypothermia produced by cannabinoid agonists. As well, *i.v., i.p*., and *p.o*. routes of CBG administration did not alter tail-flick latency (Figure 4c).

#### 3.1.3 Subsequent tetrad analyses accounting for the expected T_max_

Figure 5 displays data for catalepsy, body temperature, and the tail-flick assay according to the times when the highest blood concentrations of CBG were observed. Since there were no differences observed between sexes for CBG, the data presented includes both male and female mice (data separated by sex are presented in Supplementary Figure S3). Intraperitoneal injection of CBG (*i.p.*) produced a cataleptic response that was significant compared to vehicle *i.p.* injection or *i.v.* CBG; however, this result likely is not biologically significant as the mean was 5.4% ± 2.5% (Figure 5a). All routes of CBG administration decreased body temperature compared to vehicle, with the lowest body temperatures recorded following *i.v.* administration, followed by *i.p.* and *p.o.* routes, respectively (Figure 5b). Lastly, *i.v.* CBG administration produced an anti-nociceptive response compared to vehicle *i.v.* injection or CBG *i.p.* or *p.o.* administration (Figure 5c).

**FIGURE 6:**
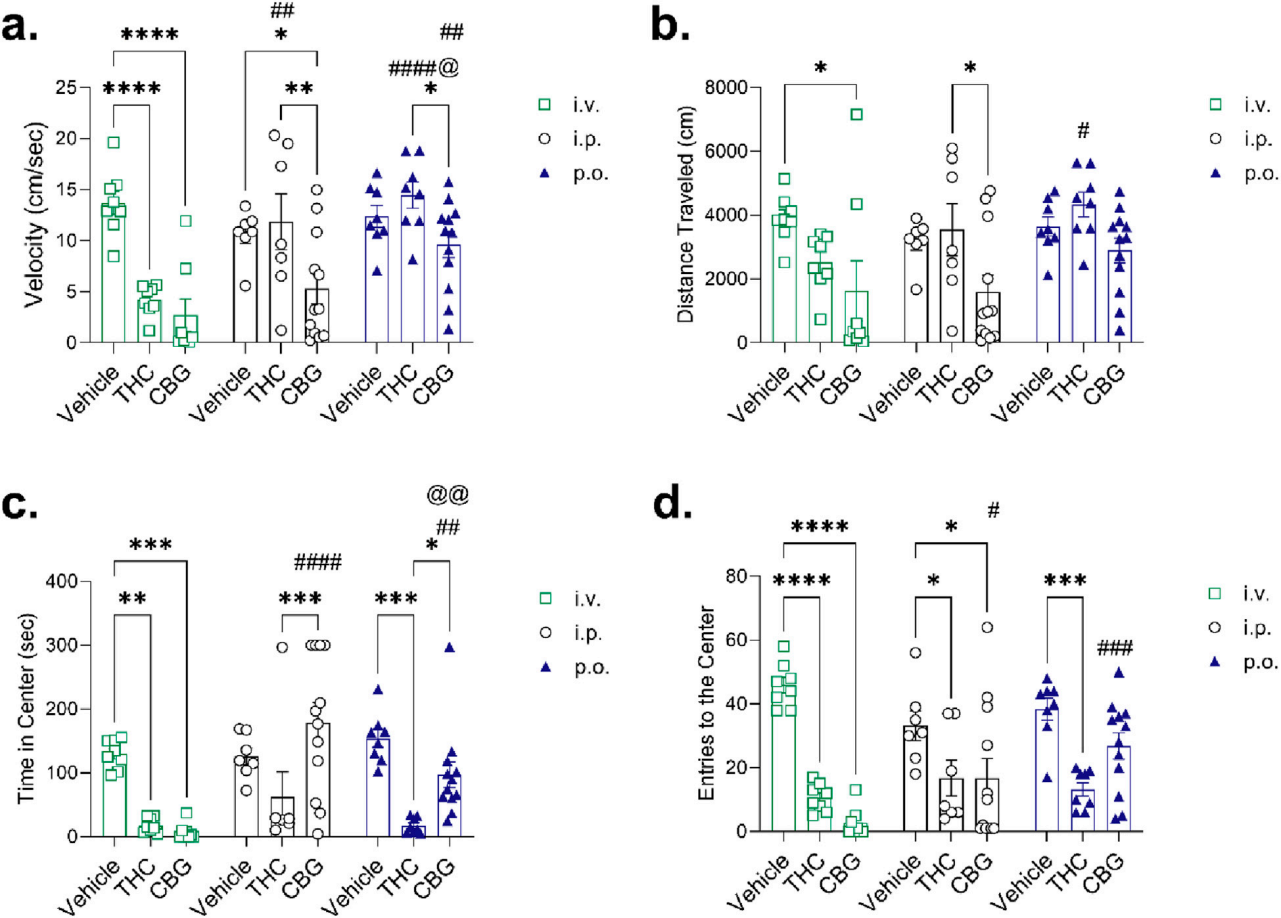
Open field test (OFT) data for male and female mice treated with 10 mg/kg THC or CBG following intravenous (*i.v.),* intraperitoneal *(i.p.)*, or oral (*p.o*.) administration (n = 7–12 per group). **(a)** Velocity (cm/sec). **(b)** Distance traveled (cm). **(c)** Time in center (sec). **(d)** Entries to the center. The OFT data were recorded 25 min post-administration for all vehicle treatments, THC given *i.v. or i.p.,* and CBG given *i.v.*; 65 min post-administration for THC given *p.o.*, 35 min post-administration for CBG given *i.p.*; and 185 min post-administration for CBG given *p.o.*. Data are expressed as mean ± SEM. ****p < 0.0001, ***p < 0.001, **p < 0.01, and *p < 0.05 within route of administration; ^####^p < 0.0001, ^###^p < 0.001, ^##^p < 0.01, and ^#^p < 0.05 compared to *i.v.* within treatment; ^@@^p < 0.01, ^@^p < 0.05 compared to *i.p.* within treatment as determined by two-way ANOVA (route *x* treatment) followed by Tukey’s *post hoc* test. *Note*, the scale of y-axes varies between panels.

To further characterize CBG, we assessed mice in the OFT considering the expected time-to-highest CBG blood concentration measured (Figure 6). Since there were no differences observed between sexes for either phytocannabinoid, the data presented includes both male and female mice (data separated by sex are presented in Supplementary Figure S4). Mice were evaluated for their velocity (Figure 6a) and distance traveled (Figure 6b) as indicators of locomotion, and time spent in the center quadrant (Figure 6c) and entries into the center quadrant (Figure 6d) as surrogates for an anxiety-modifying effect. Figure 6a illustrates that *i.v.* THC and CBG reduced velocity compared to vehicle treatment, whereas *i.p.* CBG reduced velocity compared to both *i.p.* vehicle and THC administration. As well, *p.o.* CBG reduced velocity compared to *p.o.* THC (Figure 6a). Intravenous THC reduced velocity more so than *i.p.* or *p.o.* THC; and velocity was greater in *p.o.* administered CBG compared to the *i.v.* and *i.p.* routes of administration (Figure 6a). Similar to velocity, the total distance travelled was reduced in mice administered CBG *i.v.* compared to *i.v.* vehicle and in mice administered CBG *i.p*. compared to *i.p.* THC (Figure 6b). In mice administered THC *i.v.,* movement was significantly reduced more than THC oral gavage (Figure 6b). Time in the center was reduced in mice administered either THC or CBG *i.v.* as compared to the vehicle, and in *p.o.* THC mice compared to the vehicle (Figure 6c). Mice spent significantly more time in the center of the OFT when CBG was administered by *i.p*. or *p.o.* compared to THC given *i.p.* or *p.o.* (Figure 6c). Mice were administered either *i.p.* or *p.o.* CBG spent more time in the center than mice given CBG *i.v.,* and time in the center was higher for mice administered CBG *i.p.* than mice that received CBG *p.o.* (Figure 6c). Entries to the center paralleled results for the time in the center of the OFT (Figure 6d). Mice that received *i.v.* or *i.p.* THC or CBG entered the center fewer times than their vehicle-treated counterparts, although for *i.p.* CBG mice entered the center more than mice administered CBG *i.v.* (Figure 6d). Mice that received THC via oral gavage entered the center of the OFT fewer times than vehicle oral gavage mice, and mice that received CBG *p.o.* entered the center of the OFT more times than mice given CBG *i.v.* (Figure 6d).

Overall, the data suggest that CBG, when measured at the anticipated time of highest measured blood concentrations, does evoke some minimal tetrad responses classically associated with cannabinoid receptor agonists, such as THC, although the magnitude of these effects is reduced compared to THC. Further, the magnitude of effect appears to be route-dependent, with *i.v.* administration showing the clearest responses. The differing center time and center entries observed for CBG *versus* THC in the OFT via *i.p.* and *p.o.* administration suggest CBG or its metabolites alter anxiety-like behaviours via mechanisms different to those of THC.

### 3.2 Study phase II

#### 3.2.1 Pharmacokinetic analyses

**FIGURE 7:**
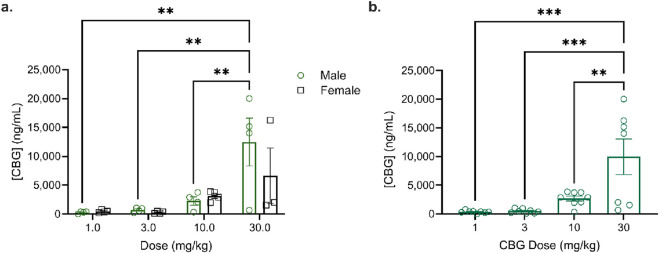
CBG blood concentrations in male and female mice aged 8–12 weeks following intravenous (*i.v.*) administration of 1, 3, 10, or 30 mg/kg CBG (*n* = 3–4/sex, *n =* 7–8/group). Blood was collected at 10 min post dose administration for each dosing level. **(a)** Data from male and female mice were displayed separately; no significant sex differences were observed. **p < 0.01 as determined by two-way ANOVA (sex *x* dose) followed by Tukey’s *post hoc* test. **(b)** Data from male and female mice were displayed together. **p < 0.01, ***p < 0.001 as determined by one-way ANOVA followed by Tukey’s *post hoc* test. Data are expressed as mean ± SEM.

Blood was drawn at 10 min from male and female C57BL/6Crl mice given 1, 3, or 10 mg/kg CBG *i.v.* to determine blood phytocannabinoid concentrations at each dose level (Figure 7). No significant difference was observed between the sexes (F (1,23) = 0.8612; p = 0.3631) (Figure 7a). At 1 and 3 mg/kg, CBG blood concentrations were similar to those we observed previously for THC (Zagzoog et al., 2024); however, at 10 mg/kg, the mean blood concentration of CBG (2,700 ng/mL) was approximately 5-times higher than that of THC (526 ng/mL) reported previously (Zagzoog et al., 2024). Supplementary Figure S3 presents the data for individual male and female mice. These data suggest that CBG blood concentrations do not scale with dose in a linear manner.

#### 3.2.2 Tetrad analyses

**FIGURE 8:**
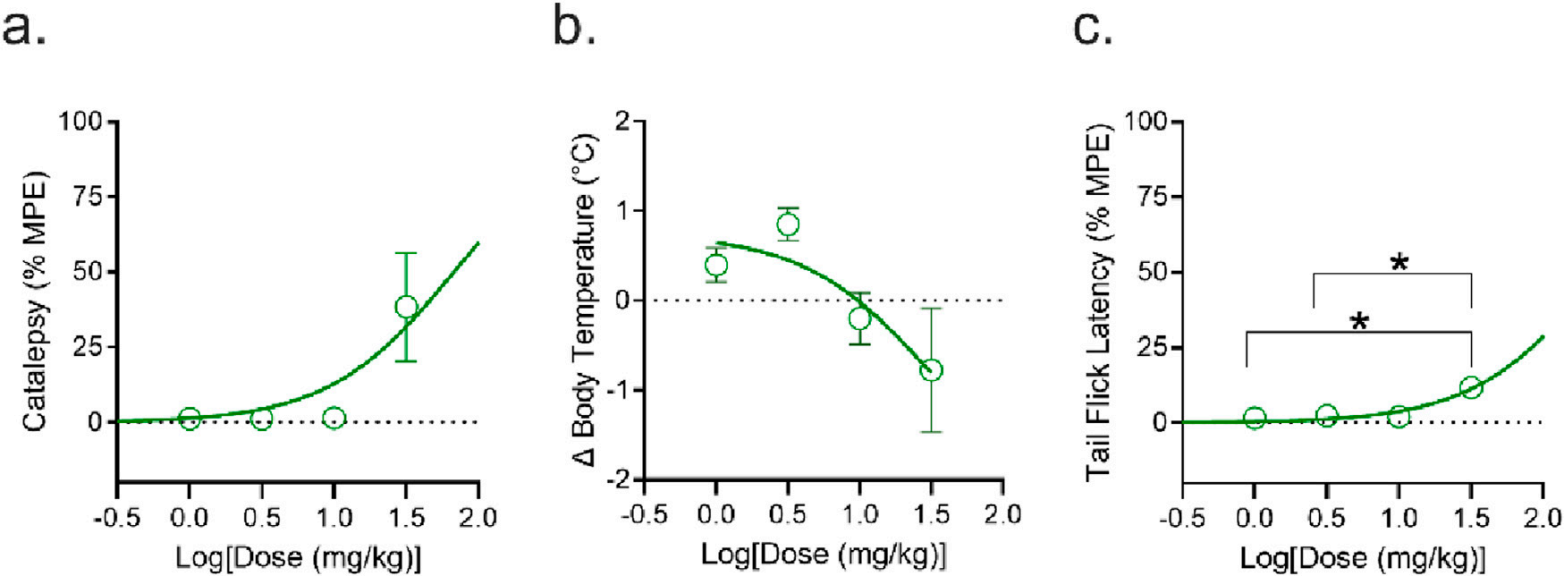
Measures of catalepsy, change in body temperature, and tail flick latency in male and female mice aged 8–12 weeks following the intravenous (*i.v.*) administration of 1, 3, 10, or 30 mg/kg CBG (n = 4 mice/dose level/sex). Mice were assessed for **(a)** catalepsy 5 min post-administration; **(b)** change in body temperature (°C) 15 min post-administration; and **(c)** anti-nociception in the tail-flick assay 20 min post-administration. Catalepsy and tail-flick latency data are expressed as the % maximum possible effect (MPE = 60 s and 20 s, respectively). All data expressed as mean ± SEM and analyzed in GraphPad (v. 10.2.3). *p < 0.05 as determined via one-way ANOVA followed by Tukey’s *post hoc* analyses.

Male and Female C57BL/6Crl mice aged 8–12 weeks were treated with varying doses of CBG (1, 3, 10, or 30 mg/kg *i.v.*). Following administration, mice were assessed for catalepsy, hypothermia, and anti-nociception (Figure 8). No significant differences were observed between the sexes; therefore, the data for male and female mice were combined (Supplementary Figure S5). For catalepsy, hypothermia, and anti-nociception, CBG produced a modest effect but only at the highest dose tested, 30 mg/kg (Figure 8a-c).

Table 2 reports blood concentrations of male mice at the first measured blood value (i.e., 10 min) (see Table 1; Figure 3) for *i.v.* administration and ED_50_ values for each physiological outcome that was estimated by fitting data to a three-parameter nonlinear regression for both male and female C57BL/6Crl mice given CBG by *i.v.* administration (Figure 8). When comparing to previously published data (Zagzoog et al., 2024) for catalepsy in the ring holding assay, CBG was 23% as intoxicating as THC. CBG did not alter body temperature and, therefore, its activity compared to THC could not be estimated. For anti-nociception, CBG was 3.6% as intoxicating as THC, when accounting for blood concentrations measured at 10 min. For all three of these outcomes, similar observations were made for both males and females (Table 2). Overall, these results confirm our findings elsewhere in the study that CBG may produce a minor degree of THC-like intoxication, but when accounting for potential pharmacokinetic differences, the effects are negligible.

**TABLE 2:** Estimated ED_50_ values for ‘intoxication’ comparisons for data presented in Figure 8 and Supplementary Figure S5.

	THC^A^	CBG	Intoxication ratio
C_at 10 min_ (ng/mL) (males)	1,290	2,843	-
ED_50_ (mg/kg) catalepsy (both sexes)	6.9	>30 [67 (36–160)]	0.23
Males	8.6	>30 [109 (42–4,400)]	0.17
Females	6.0	>30 [47 (20–170)]	0.28
ED_50_ (mg/kg) body temperature (both sexes)	7.8	>30	c.n.d
Males	8.3	>30	c.n.d
Females	7.3	n.c.	c.n.d.
ED_50_ (mg/kg) tail-flick latency (both sexes)	4.1	>30 [250 (190–240)]	0.036
Males	4.9	>30 [310 (210–570)]	0.051
Females	3.6	>30 [210 (150–320)]	0.038

C_at 10 min_ refers to the blood concentration measured 10 min post-administration. ED_50_ values for each physiological outcome were estimated by fitting data to a three-parameter nonlinear regression for both male and female C57BL/6Crl mice administered CBG intravenously (*i.v.*) (Figure 8; Supplementary Figure S5). ED_50_ data are reported to two significant digits. Intoxication ratios were estimated as described in the Methods. For data combining male and female mice, estimates of potency (ED_50_ values) was estimated by fitting data to a three-parameter nonlinear regression equation using GraphPad Prism. If estimates exceeded the dose range used in this study their estimates are reported in square brackets so that equivalency estimates could be made; otherwise, estimates of potency are stated as > 30 mg/kg for male and female data. n. c., not converged, c. n.d., could not determine. ^A^THC data were presented previously in Zagzoog et al., 2024 and shown here without error for comparison only.

## 4 Discussion

The objective of this study was to assess whether CBG was intoxicating while accounting for the CBG’s PK properties. We hypothesized that CBG would produce an intoxicating effect of lower magnitude than THC. Concordant with this hypothesis, the current findings demonstrated that CBG produced little to no intoxicating effects. CBG produced some THC-like behavioral effects at higher blood concentrations and when the compound was tested at the highest measured peak blood concentrations. However, these were generally small changes consistent with previous reports (Borrelli et al., 2013). Both our data and other studies have observed CBG’s anti-nociceptive and hypo-locomotive effects (Zagzoog et al., 2020; Schwarz et al., 2024). Our data suggests that cannabis products containing high levels of CBG are unlikely to induce intoxication at a magnitude paralleling THC. To some extent, these findings support the previously held notion that CBG is a non-psychoactive compound (Jastrząb et al., 2022).

The PK evaluations in Phase I of our study identified notable differences in the absorption kinetics of CBG depending on the route of administration, but similar disposition kinetics as exemplified by similar parameter estimates for Cl_S_, V_d_, k, and half-lives regardless of dose size. Like THC, CBG displayed two-compartmental kinetics, suggesting CBG’s distribution kinetics are similar to THC, likely due to its lipophilicity and increased tendency to partition into adipose tissues. CBG presented slower rates of absorption as indicated by the differences in timing for CBG’s highest measured blood concentrations at 30 min and 180 min for *i.p.* and *p.o.*, respectively. Notably, CBG’s bioavailability was also low, indicating poor absorption of the compound following oral gavage. In this study, CBG administered *i.p.* had a lower bioavailability (0.4) compared to previous observations with THC (F = 0.6) despite having a 1.5-fold higher C_max_ than THC (Zagzoog et al., 2024). Low CBG blood levels following *p.o.* administration suggest extensive first-pass metabolism by the gastrointestinal tract and liver. Moreover, many values for CBG in blood following *p.o.* administration fell below our assay’s LLOQ, and consequently absorption data should be interpreted cautiously. Thus, limited data points were available for analysis of *p.o.* administration data, and the only PK parameter that could be estimated was C_max_. These findings are congruent with Deiana et al. (2012), who reported CBG administration *p.o.* yielded considerably lower blood concentrations than *i.p.* CBG administration. To address this limitation, future studies could incorporate CBG deuterium standards that were not commercially available when this work was conducted, and should increase the number of serial sampling times and improve the sensitivity of the LC-MS/MS analytical method.

All PK data was collected from male mice, and the absence of female mice data represents a limitation of the current study. Although direct studies on sex differences in CBG PK in rodents are lacking, research on other cannabinoids suggests that female rodents generally may have higher absorption rates, a larger volume of distribution, and lower systemic clearance values for cannabinoid compounds compared to males (Craft et al., 2013). Female rodents may be more sensitive to cannabinoid effects, which may stem from sexual dimorphism of the endocannabinoid system, drug metabolism, and/or body fat composition (Craft et al., 2013). Moreover, cannabinoids are likely to distribute differently in males *versus* females based on body fat composition, and additional work is required to quantify cannabinoids not only in blood, but in brain, liver, and adipose tissues, where cannabinoids are likely to accumulate. Although we detected statistical significance between groups in our study, group sizes–for both males and females–could also be increased in future studies that will utilize more sensitive behavioral assays (e.g., elevated plus maze, sociability tests) to better characterize the activity of CBG. Future studies should incorporate both male and female mice, expecting a sex difference, and interrogate this difference further to determine the underlying variables that account for sex differences in cannabinoid PD and PK.

Our findings align with our previous publication, where *i.p.* CBG evoked an anti-nociceptive effect in male mice at 15 min post-injection (Zagzoog et al., 2020). The present study observed a significant anti-nociceptive effect with CBG given *i.p.* (10% ± 0.83%). Schwarz et al. reported that a 100 mg/kg *i.p.* CBG dose to male and female CD-1 mice resulted in hypothermia (30 min post-injection) and anti-nociception (20 min post-injection); however, this response did not present at lower doses (10 mg/kg) or higher doses (320 mg/kg) (Schwarz et al., 2024). Our past and current studies, as well as others’ work, clearly show the importance of timing of tetrad measurements post administration (Zagzoog et al., 2020; Zagzoog et al., 2024; Schwarz et al., 2024). Notably, responses to CBG were consistent across routes of administration despite large differences in blood concentration. This observation may indicate CBG behaves as a partial agonist of an as of yet unidentified receptor. Furthermore, it is not known whether the observed anti-nociceptive effect of CBG is mediated by cannabinoid receptors.

Significant differences in the OFT were observed depending upon the route of administration and timing of the evaluation. The efficacy of CBG administered *p.o.* may be attributed to brain disposition of CBG metabolites rather than CBG, given the especially low CBG blood levels following oral gavage reported in this study. We have previously observed reduced locomotion and increased time in the center following administration of THC *i.p.*, and no change in locomotion with increased time in the center following CBG given *i.p.* (Zagzoog et al., 2020). The differences in results between our past study are likely due to timing (1 h in Zagzoog et al., 2020 *versus* 25 min or 35 min in the current study), again emphasizing the importance of timing in these assays. Further, the observation that *i.v.* administration of CBG reduced locomotion and time in the center of the OFT compared to *i.p.* and *p.o.* administration, supporting the idea that a long-lasting metabolite of CBG may mediate the changes in OFT activity. CBG is oxidized to cyclo-CBG as a primary metabolite, which has shown bioactivity in reducing inflammation in BV2 microglial cells (Roy et al., 2022). Jenkins et al. (2023) tested minor phytocannabinoids (including CBG) for potential anxiolytic effects in male Sprague Dawley rats. Their findings indicate that acute administration of oral CBG at a dose of 100 mg/kg did not produce anxiolytic effects (Jenkins et al., 2023), which aligns with our findings using 10 mg/kg CBG compared to vehicle. However, their assessment was limited to 30 min post-administration, whereas we observed the highest levels of CBG given *p.o.* 180 min after administration. A survey or analysis of prospective CBG metabolites in mouse tissues was beyond the scope of the present study, but may be critical to future work probing the mechanism(s) of CBG’s activity.

The culmination of our study was to compare the dose-dependent effects of administered *i.v.* administration of CBG on catalepsy, body temperature, and nociception, and relate this to blood levels of CBG (Figures 7, 8). CBG did not produce an intoxication akin to THC’s effect, and we propose that any observed effects (e.g., changes in the OFT) might be the result of CBG metabolites. Given our observations of acute oral CBG administration relative to THC in the OFT, future research should assess the PD of CBG and CBG metabolites in both acute and chronic treatment models to determine the mechanism of action, magnitude, and duration of action. Acute dosing helps to understand the PK characteristics in mice to inform dosing requirements for future studies evaluating chronic dosing scenarios. This was a necessary first step to ongoing PK and PD evaluations of CBG. That our study strictly examined acute administration is a limitation of the current work, but it represents an important first step toward more translational future models, including chronic administration.

A growing interest in CBG for its potential medicinal benefits has resulted in a number of ongoing clinical studies investigating its therapeutic potential in a range of conditions, including pain, inflammation, and neurodegenerative diseases. A recent human clinical study published by Cuttler et al. (2024) investigated the acute effects of CBG on mood, anxiety, stress, and cognitive function (Cuttler et al., 2024). The study utilized a subjective state rating of anxiety and reported a 26.5% reduction in anxiety levels from baseline following oral administration of 20 mg CBG (Cuttler et al., 2024). Additionally, a prior survey study indicated that 51% of CBG users utilize it to manage anxiety, with 78% of these individuals finding it more effective than conventional anxiety medications (Russo et al., 2022). This body of evidence underscores the importance of CBG in the field of clinical research, highlighting its potential as a therapeutic agent. As more clinical trials are conducted, we can better understand their efficacy and safety profile, potentially leading to new, non-intoxicating treatments for various ailments.

## 5 Conclusion

In conclusion, we found that the route of administration significantly influences both CBG’s effects and blood concentrations. CBG did not produce the intoxicating properties associated with THC when accounting for route of administration, dose, sex, or blood concentration. To build on these findings, additional studies are necessary to explore the combined effects of CBG and THC as may be found in cannabis products, with a focus on their metabolism and detailed PD and PK profiles.

## Data Availability

The original contributions presented in the study are included in the article/Supplementary Material, further inquiries can be directed to the corresponding author.
