# Supplementary material for: Pharmacokinetics and pharmacodynamics of cannabigerol (CBG) in the C57BL/6Crl mouse
Source: Front Pharmacol. 2025 Dec 9;16:1672098. doi: 10.3389/fphar.2025.1672098 (PMC12722920; doi:10.3389/fphar.2025.1672098)
Supplement: Supplementary file 1 [file Supplementaryfile1.docx]

Supplemental Data for: Pharmacokinetics and Pharmacodynamics of Cannabigerol (CBG) in the C57BL/6Crl Mouse

# Analytical Method Results

Working stocks for standard curve and quality control (QC) concentrations were prepared by diluting 1 mg/mL CBG in methanol. Working standards were made in the range of 160-8000 ng/mL, and QC’s working solutions were 160 ng/mL, 480 ng/mL, 4,000 ng/mL, and 5,500 ng/mL to give a lower limit of quantification (LLOQ), low-quality control (LQC), middle-quality control (MQC), and high-quality control (HQC) of 8 ng/mL, 24 ng/mL, 200 ng/mL, and 275 ng/mL respectively. Standards and QCs were prepared by adding 5 μL of working standard or working QC solution to 95 μL of blank mouse whole blood and mixing gently for 10 sec. To each standard or QC, 300 μL of super mix internal standard solution (acetonitrile containing 0.5% formic acid and 15 ng/mL CBD-d3) was added. Each standard or QC was vortexed for 30 sec, then centrifuged at 14,000 × g at 4°C for 10 min using an Eppendorf 5415R centrifuge. The supernatant was transferred to a HybridSPE®-Phospholipid 96-well plate, filtered through a low vacuum, and then transferred to an amber glass HPLC vial.

Mouse whole blood samples were then thawed at room temperature and gently mixed via inversion. 300 μL of super mix (acetonitrile containing 0.5% formic acid and 15 ng/mL CBD-d3) internal standard solution was added to 100 μL of a whole blood sample, and the above protocol was followed. HPLC-MS/MS was used to quantify CBG and THC in mouse whole blood using a protocol described and performed in (Zagzoog et al., 2024).

# HPLC-MS/MS Validation Method

Matrix effects, selectivity, carry-over, linearity, precision, accuracy, recovery, and reproducibility were all taken into consideration during the method validation, which was done in accordance with FDA and EMA Guidance for Bioanalytical Method Validation. To ensure selectivity (**Supplementary** **Figure S1**), blank whole blood from different mice was tested for interference in CBG MRMs, with acceptance criteria set at an interference of less than 20% of the LLOQ.

The matrix effect was assessed by evaluating suppression or enhancement of co-eluting compounds on the response of the analyte of interest using the low, medium, and high QC samples. Each QC concentration was performed in replicates of six and calculated by Eq S1. All levels of QC samples were spiked post-extraction and compared to the analyte in pure solvent. In order to assess extraction recovery and how much analyte is lost during processing, QC samples were spiked before extraction, and another set was spiked following extraction. By analyzing the peak areas for each concentration obtained from the pre- and post-spiked sets, the recovery was determined using Eq. S2.

**Eq. S1** $Matrix Factor \left( \mathrm{MF} \right)= \frac{{Area}_{post-spike}}{{Area}_{pure}}$

**Eq. S2** $Extraction Recovery \left( \% \right)= \frac{\mathrm{Area}_{Pre-Spike}}{\mathrm{Area}_{Post-Spike}} \times100$

Carry-over was examined to determine analyte or internal standard transfer from one sample to another, which would result in overestimation of sample analyte concentrations. The carry-over effect was calculated by comparing the peak area ratio of the blank sample to the LLOQ sample immediately following the processing of the ULOQ (upper limit of quantification) using Eqs. S3 and S4 for the standard and internal standard, respectively. No significant carry-over effect was considered if the area ratio was equal to or less than 20% of the LLOQ and less than 5% for the internal standard. Peak-area ratios (peak area ratio of the analyte to internal standard) versus analyte concentration were used to construct a standard curve with 8 points ranging from 8 ng/mL to 400 ng/mL (8 ng/mL, 15 ng/mL, 20 ng/mL, 50 ng/mL, 150 ng/mL, 300 ng/mL, 350 ng/mL, and 400 ng/mL).

A linear regression analysis of the standard curve was weighted with 1/X^2^, and accuracy, slope, intercept, and coefficient of determination assessed the performance of the standard curve over three separate occasions as per FDA guidelines.

**Eq. S3** $Carry Over \left( \mathrm{St} \right)= \frac{Area Blank after ULOQ}{Area LLOQ} \times100$

**Eq. S4** $Carry Over \left( \mathrm{IS} \right)= \frac{Area Blank after ULOQ}{Area LLOQ} \times100$

# Analytical Method Validation Results

Intra-day and inter-day accuracy and precision was assessed using the LLOQ, LQC, MQC, and HQC in replicates of six over three separate runs on three different analytical days (**Supplementary** **Table S1**). Precision was recorded as CV%, and no QC level exceeded 8%. Accuracy was expressed as a percentage of the theoretical value and ranged from 98% to 112% (**Supplementary** **Table S1**). On three days of analysis, inter-day accuracy and precision were validated using 18 repetitions for the LLOQ, LQC, and MCQ and 16 replicates for the HCQ (**Supplementary** **Table S2**). Accuracy values ranged from 100 to 111%, and precision values ranged from 4.5 to 4.9% (**Supplementary** **Table S2**). Precision and accuracy values for the LQC, MQC, and HQC must be within 100 ± 15%, while the LLOQ must be 100 ± 20% in order to meet FDA acceptability criteria. All intra-day and inter-day accuracy and precision data met these requirements.

Selectivity for the standard and internal standard on all days of analysis was found to be < 20% or < 5%, respectively, meeting FDA standards. A chromatogram for double blank showed no interfering endogenous peaks at the same retention time for both standard and internal standard (**Supplementary** **Figure S1)**. Matrix factors were evaluated for the LQC, MQC, and HQC and found to be 102%, 110%, and 113%, respectively, for the standard and 109%, 109%, and 115% for the internal standard. These meet the FDA acceptability requirement of < 15% (**Supplementary** **Table S3).**

LQC, MQC, and HQC (24 ng/mL, 200 ng/mL, and 275 ng/mL, respectively) recovery was ~70% (**Supplementary** **Table S3**). Limiting carry-over to a minimum ensures that the analysis of subsequent samples is not impacted by earlier samples. The carry-over effect evaluated on 3 separate days was found to be 1.5% to 2.5% for the CBG standard, well within FDA guidelines of 20%.

Linear regression analysis of the peak-area ratios against nominal concentrations of the 8-point standard curve performed on 3 separate days established linearity with R^2^ values of 0.9961 – 0.9996 and no significant differences in the intercept values (**Supplementary** **Figure S2)**.

**Figure and Legends for Figures**


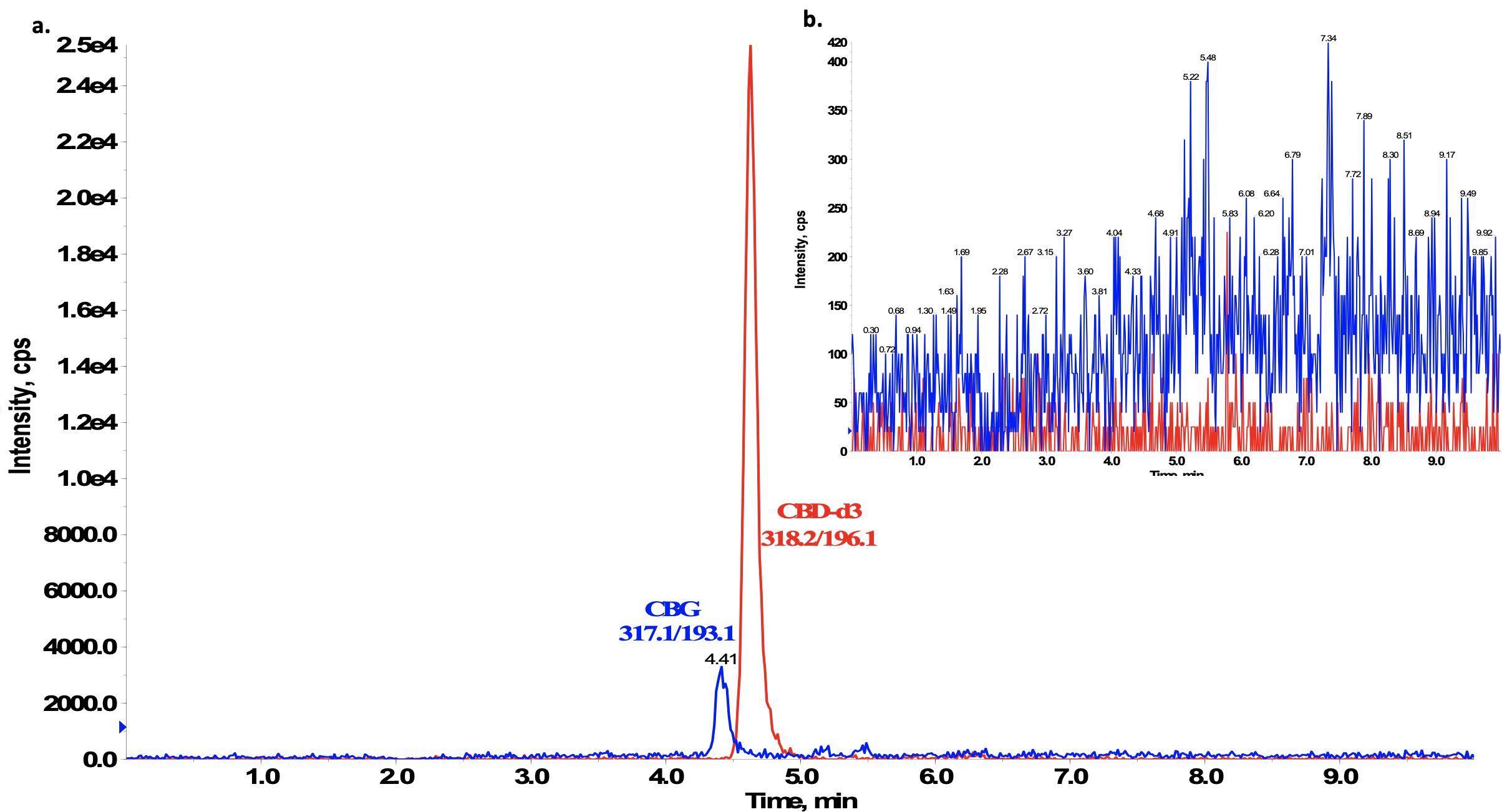


**Figure S1.** Representative LC-MS chromatogram of (a) blank and (b) double blank sample of mouse whole blood spiked with 8 ng/mL of CBG, and 15 ng/mL of internal standard (IS) (CBD-d3). *Note,* y-axes vary between panels to better display the data.

**Figure S2.** CBG standard curves were performed on 3 separate days. Standard curves were linear with R^2^ values ≥ 0.98. Eight standard points, ranging from 8 ng/mL to 400 ng/mL, were used to generate standard curves with weighting at 1/X^2^.


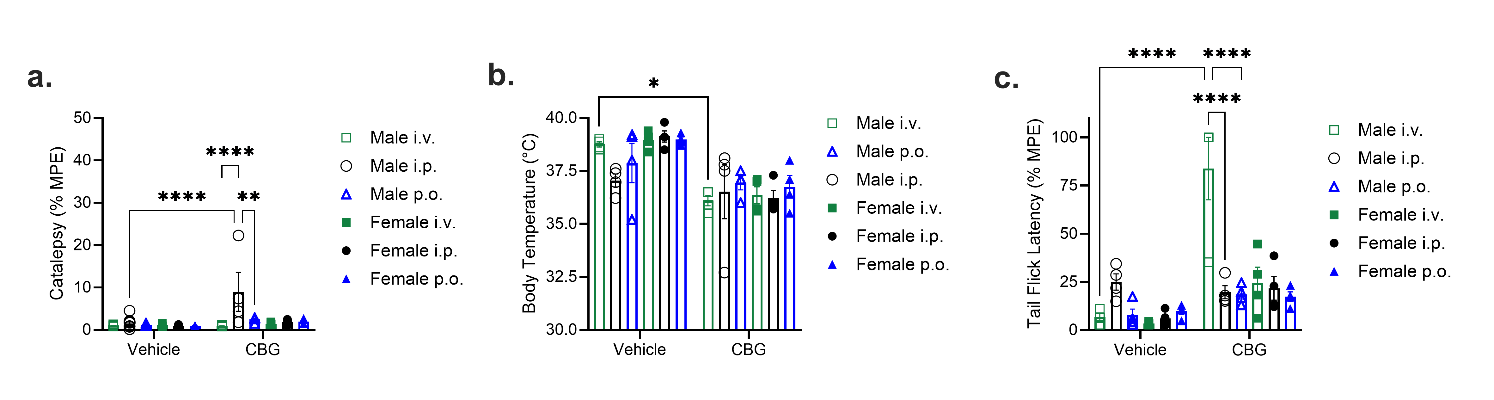


**Figure S3**. Physiological effect of 10 mg/kg of CBG in male and female C57BL/6Crl mice. Data are presented for each sex for each route of administration, intravenous (*i.v.*), intraperitoneal (*i.p.*), or oral (*p.o.*)*.* (**a)** Catalepsy time was 5 min post-injection for *i.v.* CBG; 20 min post-injection for *i.p.* CBG; and 170 min post-injection for *p.o.* CBG. (**b**) Body temperature time was 15 min post-injection for *i.v.* CBG; 25 min post-injection for *i.p.* CBG; and 175 min post-injection for *p.o.* CBG (dotted line represents 37.5°C). (**c**) Nociception in the tail-flick latency test was 20 min post-injection for *i.v.* CBG; 30 min post-injection for *i.p.* CBG; and 180 min post-injection for *p.o.* CBG. Data for catalepsy are represented as the % maximum possible effect (MPE) during a maximum of 60 sec. Body temperature data are expressed as the measured body temperature via a rectal thermometer (°C). Tail-flick latency data are expressed as the % MPE during a maximum of 20 sec. n = 4 - 12 animals per treatment group. Data are expressed as mean ± SEM. ****p<0.0001, **p < 0.01, and *p < 0.05 as determined by two-way ANOVA (route *x* treatment) followed by Tukey’s post-hoc test. *Note*, vehicle data were previously included in Zagzoog et al. (2024); and the scale of y-axes varies between panels. Corresponding data for both sexes are presented in **Figure 5.**


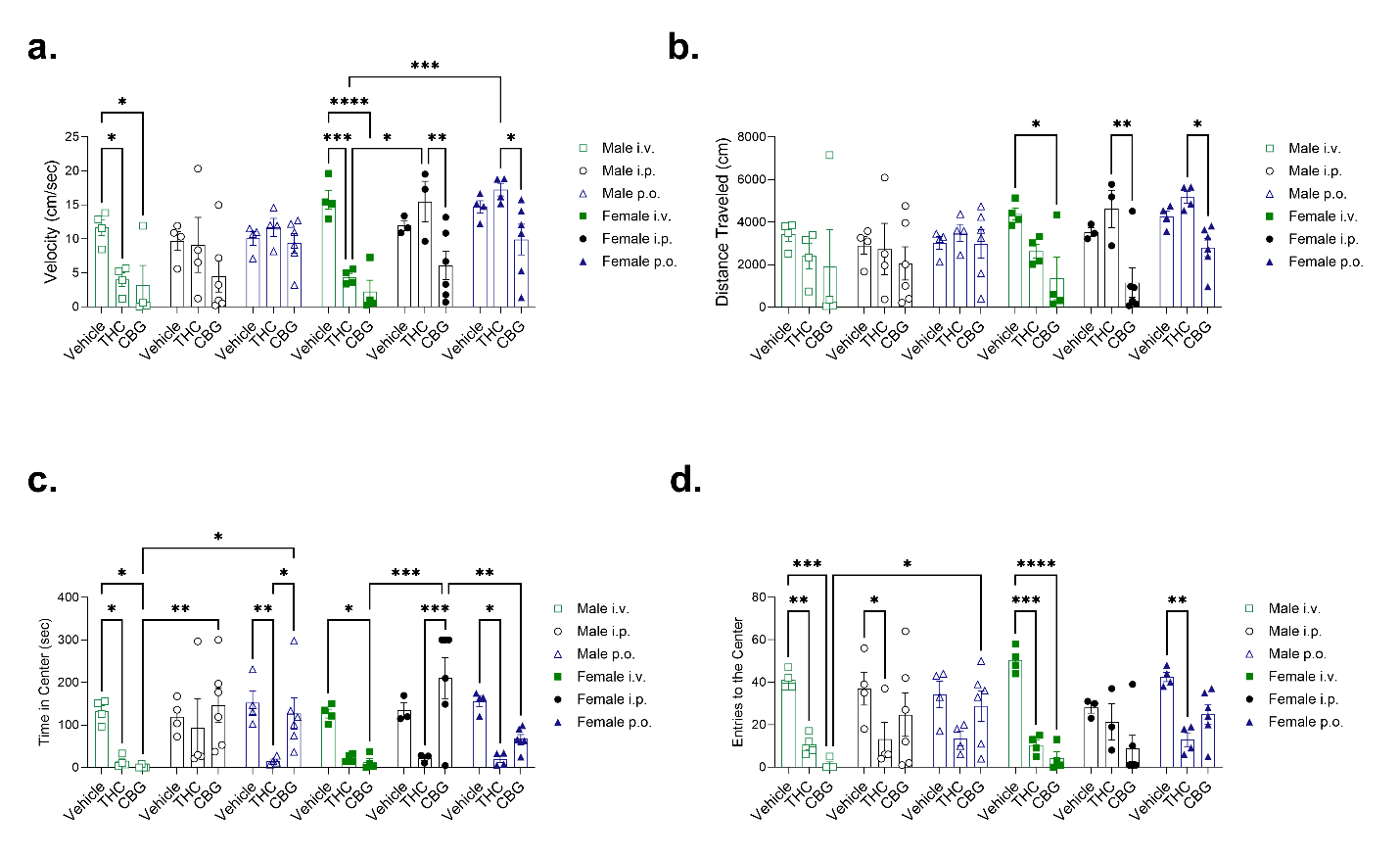


**Figure S4**. Open field test (OFT) data for male and female mice treated with 10 mg/kg THC or CBG intravenous (*i.v.*), intraperitoneal (*i.p.*), or oral (*p.o.*). *(***a**) Velocity (cm/sec). (**b**) Distance traveled (cm). (**c**) Time in center (sec). (**d**) Entries to the center. The OFT data were recorded 25 min post-injection for all vehicle treatments, *i.v., i.p.* THC and *i.v.,* CBG; 65 min post-injection for *p.o.* THC, 35 min post-injection for *i.p.* CBG; and 185 min post-injection for *p.o.* CBG. n = 3 - 6 animals per treatment group. Data are expressed as mean ± SEM. ****p < 0.0001, ***p<0.001, **p < 0.01, and *p < 0.05 as displayed and determined by two-way ANOVA (route *x* treatment and sex) followed by Tukey’s post-hoc test. *Note* that the scale of y-axes varies between panels. Corresponding data separated by both sexes are presented in **Figure 6.**


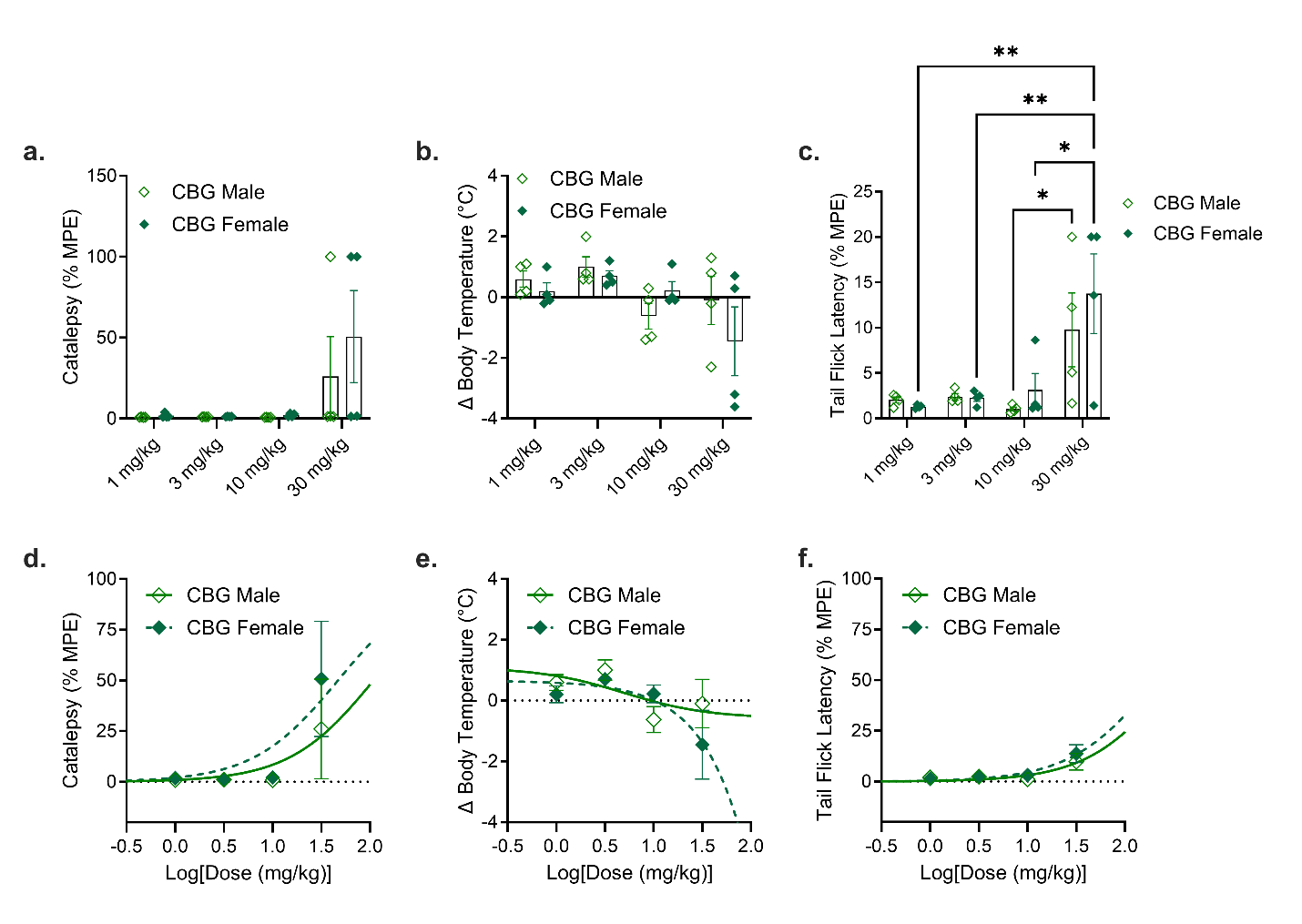


**Figure S5.** Male and female mice aged 8-12 weeks were treated with 1, 3, 10, or 30 mg/kg intravenous (*i.v.*) CBG. Animals were assessed for: (**a,d**) catalepsy 5 min post-injection; (**b,e**) change in body temperature 15 min post-injection; and (**c,f**) anti-nociception in the tail flick assay 20 min post-injection. Catalepsy and tail flick latency data are expressed as the % maximum possible effect (MPE = 60 sec and 20 sec, respectively). All data was expressed as mean ± SEM and analyzed in GraphPad (v. 10.2.3). n = 4 animals per treatment group. *p<0.05, **p<0.01 as determined by two-way ANOVA (sex *x* dose) followed by Tukey’s post-hoc analyses. Corresponding data separated by both sexes are presented in **Figure 8.**

**Tables**

**Table S1.** Intraday accuracy and precision for CBG in mouse whole blood quantified using LC-MS/MS.

| **Quality control** | **Replicates** | **Analysis day (#)** | **Observed concentration (mean ± SD; ng/mL)** | **Precision (CV%)** | **Accuracy (%)** |
| --- | --- | --- | --- | --- | --- |
| **LLOQ** | 6 | 1 | 8.5 ± 0.51 | 5.8 | 109.3 |
|  | 6 | 2 | 8.6 ± 0.37 | 4.3 | 107.8 |
|  | 6 | 3 | 8.5 ± 0.35 | 4.5 | 106.1 |
| **LQC** | 6 | 1 | 24.6 ± 1.48 | 6.0 | 102.7 |
|  | 6 | 2 | 25.5 ± 0.74 | 3.0 | 106.2 |
|  | 6 | 3 | 25.8 ± 1.27 | 4.9 | 107.7 |
| **MQC** | 6 | 1 | 195.7 ± 10.80 | 5.5 | 97.9 |
|  | 6 | 2 | 204.7 ± 9.27 | 4.5 | 102.4 |
|  | 6 | 3 | 203.7 ± 4.97 | 2.4 | 101.7 |
| **HQC** | 6 | 1 | 302.5 ± 17.48 | 7.8 | 109.8 |
|  | 5 | 2 | 291.8 ± 15.32 | 5.2 | 110.8 |
|  | 5 | 3 | 309.7 ± 9.0 | 2.9 | 112.5 |

LLQC, lowest limit of quantification (8 ng/mL); LQC, low quality control (24 ng/mL); MQC, medium quality control (200 ng/mL); HQC, high quality control (275 ng/mL).

**Table S2.** Interday accuracy and precision for CBG in mouse whole blood quantified using LC-MS/MS.

| **Quality control** | **Replicates** | **Observed concentration (mean ± SD; ng/mL)** | **Precision (CV%)** | **Accuracy (%)** |
| --- | --- | --- | --- | --- |
| **LLOQ** | 18 | 8.6 ± 0.4 | 4.67 | 107.7 |
| **LQC** | 18 | 25.3 ± 1.24 | 4.92 | 105.5 |
| **MQC** | 18 | 201.3 ± 9.17 | 4.55 | 100.7 |
| **HQC** | 16 | 306.1 ± 13.94 | 4.55 | 111.2 |

LLQC, lowest limit of quantification (8 ng/mL); LQC, low quality control (24 ng/mL); MQC, medium quality control (200 ng/mL); HQC, high quality control (275 ng/mL)

**Table S3.** Extraction efficacy, recovery, and matrix factor for CBG standard and internal standard in mouse whole blood quantified using LC-MS/MS.

| Quality control | Standard Extraction Efficacy % | Standard Recovery % | Standard Matrix Factor % | Internal Standard  Extraction Efficacy % | Internal Standard Recovery % | Internal Standard  Matrix Factor % |
| --- | --- | --- | --- | --- | --- | --- |
| LQC | 68.0 ± 3.8 | 69.4 ± 8.3 | 102.3 ± 13.2 | 114.8 ± 10.6 | 123.9 ± 7.9 | 109.2 ± 17.0 |
| MQC | 61.6 ± 6.4 | 67.6 ± 7.0 | 110.0± 7.9 | 111.8 ± 2.4 | 122.1 ± 10.3 | 109.3 ± 9.1 |
| HQC | 60.1 ± 5.0 | 67.7 ±8.4 | 113.1±14.8 | 105.1± 9.4 | 120.9 ± 11.1 | 115.2 ± 6.3 |

LQC, low quality control (24 ng/mL); MQC, medium quality control (200 ng/mL); HQC, high quality control (275 ng/mL).

**
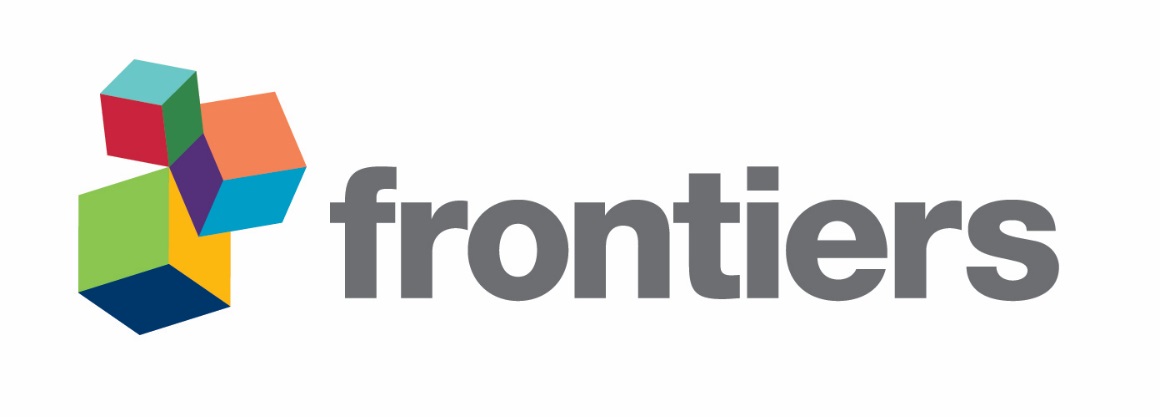
**
